# Differential diagnosis of eccrine spiradenoma: A case report

**DOI:** 10.3892/etm.2014.1906

**Published:** 2014-08-14

**Authors:** YAN ZHENG, QIONG TIAN, JUAN WANG, XINYU DONG, HUILING JING, XIN WANG, YIGUO FENG, SHENGXIANG XIAO

**Affiliations:** Department of Dermatology, The Second Affiliated Hospital of Xi’an Jiaotong University, Xi’an, Shaanxi 710004, P.R. China

**Keywords:** eccrine spiradenoma, differential diagnosis, immunohistochemistry

## Abstract

Eccrine spiradenoma (ES) is a rare, benign adnexal neoplasm that may easily be mistaken for glomus lesions or angioleiomyoma due to its painfulness and florid vascularization. A 44-year-old male with a blue-colored, nodular tumor on the left knee, present for 10 years, was submitted for diagnosis. Dermatological examination was undertaken, followed by surgical excision of the subcutaneous lesion and histopathological examination of the tissue. Subjective symptoms included tenderness upon palpation and routine investigations were within normal limits. Immunohistochemical analysis of the tumor cells demonstrated positive staining for CK5/CK6, CK8/CK18, S100, as well as small vacuole-like positive for EMA, and was therefore diagnosed as ES. The results of the present study suggest that immunohistochemical assays may be helpful to clarify the diagnosis and differentiate ES from other painful subcutaneous tumors exhibiting similar clinical and histological presentations.

## Introduction

Eccrine spiradenoma (ES) is a rare, benign adnexal neoplasm that has been historically designated as a tumor of eccrine differentiation. ES is able to be present on any part of the body (1), with ~1/5 cases occurring in the extremities (2). ES can appear at any age, and no gender predominance has been reported. The treatment of choice of ES is surgical excision with clear margins, while recurrence has been documented in the literature (3). Malignant transformation of ES is rare, but malignant ES is quite aggressive and can occur within a long-standing lesion that makes the early definitive diagnosis of ES of major importance. ES may easily be mistaken for glomus lesions or angioleiomyoma due to its painfulness and florid vascularization. In the current case study, a noteworthy case of ES in the left knee is presented, with focus upon its clinical presentation, histopathological characteristics and differential diagnosis from other painful subcutaneous tumors that exhibit a similarly high degree of vascularization.

## Case report

### Case summary

**Figure 1:**
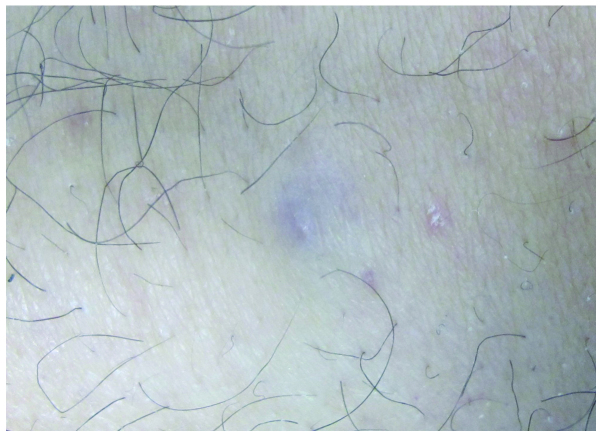
Overall, the lesion was a firm, tender, well-defined, blue nodule with a smooth surface and obscure boundaries. Stromal infiltration was evident without epidermal connections.

A 44-year-old male presented with a blue intradermal nodule ~1 cm in size localized in the left knee. The tumor was initially observed 10 years previously without any associated pain or pruritus and gradually enlarged thereafter. Dermatological examination revealed a firm, tender and blue nodule with a smooth surface and obscure boundaries (Fig. 1). Stromal infiltration was evident without epidermal connections. Routine investigations were within normal limits and the patient revealed no other significant past medical or family history. Surgical excision of the subcutaneous lesion was performed and the tissue was submitted for microscopic examination. The patient was treated by a local, complete excision without recurrence 16 months later. The study was approved by the Second Affiliated Hospital of Xi’an Jiaotong University (Xi’an, China) and written informed consent was obtained from the patient.

### Histopathological examination

**Figure 2:**
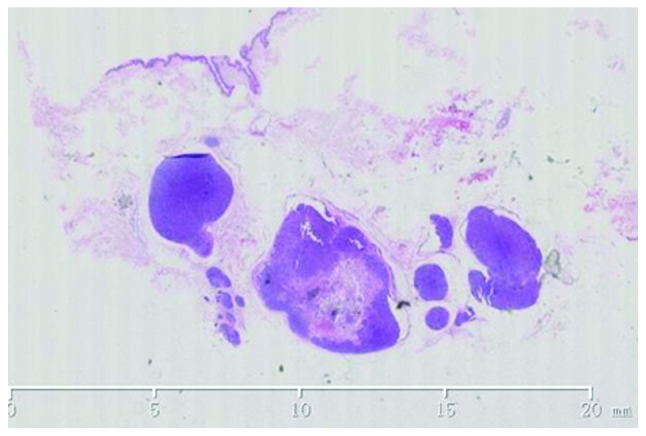
Histological examination revealed multiple strongly basophilic lobules arranged in sheets in the dermal and subcutaneous tissue. The overlying epidermis was almost intact without connections to the tumor island. Bar length, 10 mm.

**Figure 3:**
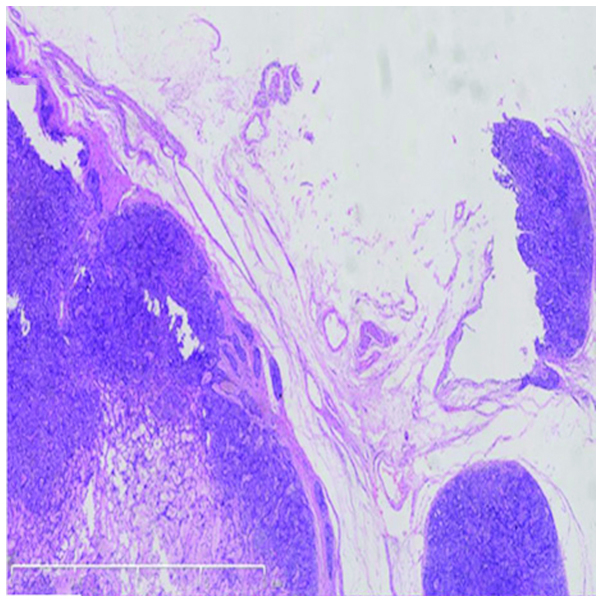
The nodule was well marginated and encapsulated by a thick and dense fibrous capsule. Bar length, 2 mm.

**Figure 4:**
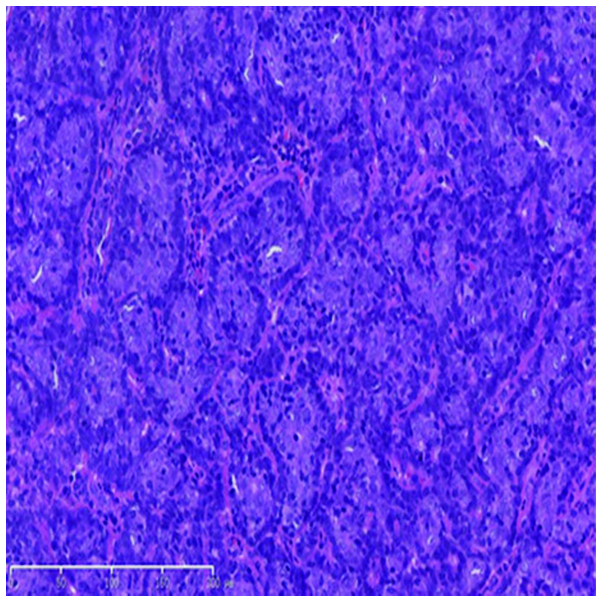
Two types of tumor cells were recognized in the lobules: small, darkly stained basaloid cells located at the periphery and larger cells with pale and acidophilic nuclei situated mainly in the center. Bar length, Bar length, 200 μm.

**Figure 5:**
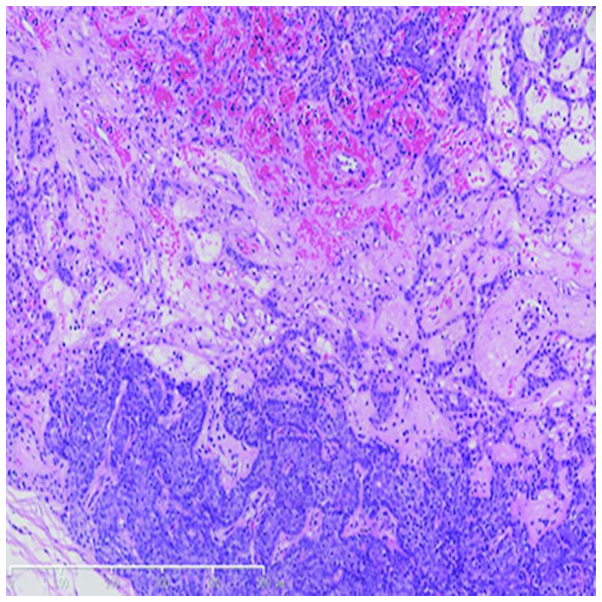
Tumor cells with tubular differentiation, lymphocyte infiltration and abundant telangiectasia were identified; however, these did not reveal significant mitosis. Bar length, 500 μm.

**Figure 6:**
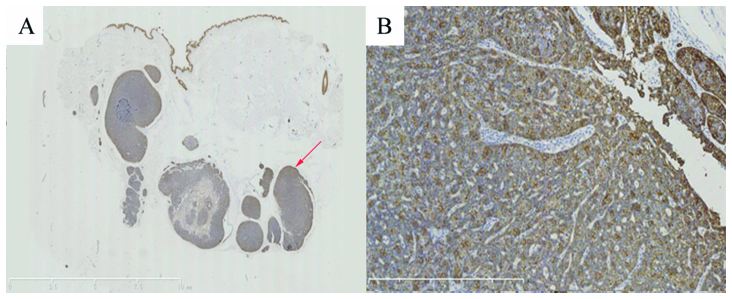
(A) Immunohistochemical staining of the tumors revealed positive immunoreactions for cytokeratin (CK)5/CK6. (B) Enlarged tumor detail from the section indicated by the red arrow. Bar length: (A) 10 mm and (B) 200 μm.

**Figure 7:**
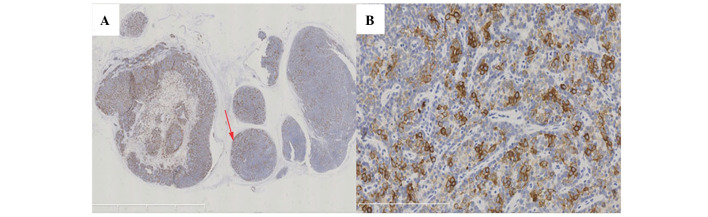
(A) Positive reaction of tumor staining with cytokeratin (CK)8/CK18 antibodies. The glandular epithelial cells in the small mass were also positively stained. (B) Enlarged tumor detail from the section indicated by the red arrow. Bar length: (A) 4 mm and (B) 1 mm.

**Figure 8:**
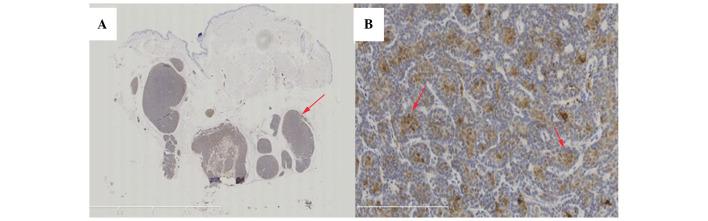
(A) S100 protein-positive cells. (B) Enlarged tumor detail from the section indicated by the red arrow in (A), with irregularly shaped nuclei (red arrow), mainly with positive cytoplasmic staining. Bar length: (A) 10 mm and (B) 500 μm

**Figure 9:**
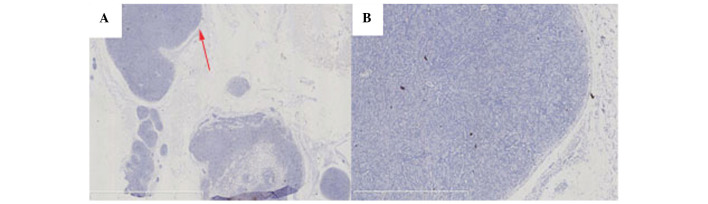
(A) Immunohistochemical staining showing negative immunoreaction for carcinoembryonic antigen. (B) Enlarged tumor detail from the section indicated by the red arrow in (A). Bar length: (A) 4 mm and (B) 1 mm.

**Figure 10:**
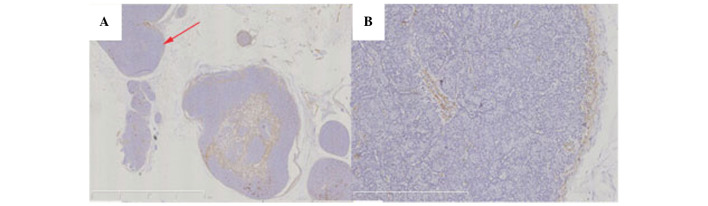
(A) Tumor cells were not stained with the smooth muscle actin (SMA) antibody. (B) Enlarged tumor detail from the section indicated by the red arrow in (A). Bar length: (A) 4 mm and (B) 1 mm.

**Figure 11:**
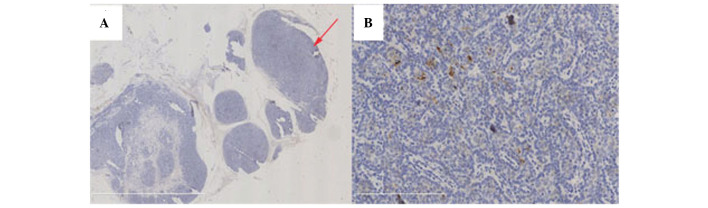
(A) Small vacuole-like positivity of the differentiated glandular epithelial cells following staining with the anti-epithelial membrane antigen antibody. (B) Enlarged tumor detail from the section indicated by the red arrow in (A).

An excisional biopsy was performed. Histological examination revealed multiple strongly basophilic lobules arranged in sheets in the dermal and subcutaneous tissue. The overlying epidermis was almost intact without connections to the tumor island (Fig. 2). The nodule was well marginated and encased by an abundant eosinophilic capsule (Fig. 3). Two types of cells were recognized in the lobules, namely small, darkly stained basaloid cells located at the periphery and larger cells with a pale and acidophilic nucleus situated mainly in the center (Fig. 4). Tumor cells were arranged irregularly into small cystic sweat gland ducts, lined with the acidophilic epithelial cells. Certain tubular differentiations were conspicuous among the tumor cells, as well as lymphocyte infiltration and abundant telangiectasia, with irregular clearance identified in the lumen. However, mitosis was not observed (Fig. 5). The immunohistochemical staining of the tumors revealed positive immunoreactions for cytokeratin (CK)5/CK6 (Fig. 6), CK8/CK18 (Fig. 7) and S100 (Fig. 8); and negative immunoreactions for carcinoembryonic antigen (CEA; Fig. 9) and smooth muscle actin (SMA) (Fig. 10). Staining with anti-endomysial antibody (EMA) revealed positive vacuole-like structures on the surfaces of the glands and intracytoplasmic lumens in certain tumor cells (Fig. 11). From these results, a diagnosis of the tumor as ES was established.

### Differential diagnosis

ES may be easily mistaken for other lesions that characteristically present with localized pain and/or a marked degree of vascularization. These include: i) aggregated lymphatic nodules; in the primary clinical differential diagnosis, the immunohistochemical results are usually clear (tubular differentiation was demonstrated in the present case); ii) glomus tumor, a benign neoplasm characteristically associated with conspicuous vasculature components (poor vasculature was observed in the current case); and iii) angioleiomyoma, a benign tumor arising from the vascular smooth muscle typically expressing SMA (which was negative in the present case).

## Discussion

Eccrine spiradenoma (ES), as first described in 1956, is a rare, benign adnexal neoplasm that is able to present on any part of the body, with ~1/5 of cases occurring in the extremities (4). The present study reported a case of ES located in the left knee. It classically presents in patients between the ages of 20 and 40 years and is primarily described as a firm or soft and spongy textured, round or ovoid-shaped and blue-colored lobulated mass, ranging in size from 0.5 to 5 cm in diameter. The most striking clinical feature of ES lesions is the presence of pain or tenderness (3); however, no excruciating pain was presented in the current case. The majority of ES presentations are solitary, with males and females being affected equally (5). The presence of concomitant cylindroma and trichoepithelioma in certain ES patients may increase the possibility of Brooke-Spiegler syndrome (6). Malignant transformation is extremely rare and generally arises from long-standing benign ES (7).

Histologically, ES may present in a variety of ways, including as tumors arranged in sheets, cords or islands, often precluding a straightforward diagnosis. Tumor cells are strongly basophilic, resembling lymph nodes when observed under a low power microscope. In certain cases, lymphocyte infiltration and abundant telangiectasia are observed in the tumor region, with irregular clearance presented around the lumen. A differential diagnosis for glomus tumors should be performed when vascular hyperplasia is statistically significant. Occasionally, a nerve trunk may be observed in the vicinity of the lobules, as identified in the present case.

The diagnosis of ES may be elusive given its multiple presentations without a change in the skin surface. Correct diagnosis is critical due to the potential for malignancy. The primarily clinical feature of ES is the presence of pain in the patient (8) and painful dermal tumors should be taken into consideration on initial evaluation. Entities including angioma, angioleiomyoma and neuroma should be considered in the further differential diagnosis of ES given their similar presentations. The diagnosis may be distinguished histologically if the clinical picture is not distinctive. However, the histological results of ES have been observed to be consistent with those of cylindroma within the same biopsy, as numerous tumors demonstrate overlapping features between the two entities (9). A previous study suggested that the two entities may represent two extremes on a continuous spectrum of dermal tumors that originate from a common progenitor (3). The histological differentiation of cylindroma and ES is less straightforward; although, with the help of pathological and immunohistochemical presentations, an improved diagnosis may be achieved. Furthermore, when the tubular differentiation of the intralobular duct cells is less significant, it may be mistaken for an aggregated lymphatic nodule. Immunohistochemical methods may be used for its differential identification.

A clinical differential diagnosis of glomus tumor/aggregated lymphatic nodule was offered in the current case. The diagnosis could not be confirmed from the clinical and histological investigations and an immunohistochemical assay was performed. A diagnosis of ES was suggested on the basis of ductal differentiation and poor vasculature identified following immunohistochemical staining of the excised tumor mass.

ES has been historically designated as a tumor of eccrine lineage, although the current view is that it may arise due to an apocrine process (3,10). In the current case, the immunophenotype of the tumor exhibited characteristic features of eccrine differentiation along with the expression of the S100 protein and CK5/CK6. Staining with an anti-EMA antibody revealed small vacuole-like positivity of the lumen surfaces, while the tumor cell staining for CEA and SMA antibodies was negative. There was no clear evidence of myoepithelial differentiation. However, tubular differentiation of tumor cells was also demonstrated, which would be expected in an apocrine neoplasm (11,12). Thus, further investigation was required.

Treatments for ES have not been well established; however, surgical excision is currently the gold standard option, with low rates of recurrence documented (3). Other treatment options, including radiotherapy, carbon dioxide laser ablation and chemotherapy, have also been proposed although no studies have substantiated an optimal practice (13). For cases of familial ES, genetic counseling has been advised (14).
